# Unusual presentation of a retrobulbar optic nerve sheath meningioma: a case report

**DOI:** 10.3389/fneur.2026.1785083

**Published:** 2026-04-23

**Authors:** Johannes Robert Fleischer, Soroush Doostkam, Wolf Lagrèze

**Affiliations:** 1 Eye Center, Medical Center, Faculty of Medicine, University of Freiburg, Freiburg, Germany; 2 Medical Center, Faculty of Medicine, Institute of Neuropathology, University of Freiburg, Freiburg, Germany

**Keywords:** biopsy, meningioma, neuroopthalmology, optic nerve sheath, retrobulbar

## Abstract

**Introduction:**

We report a case of a slowly progressive unilateral visual field defect associated with an atypical retrobulbar optic nerve sheath lesion in the context of concurrent breast cancer, which was ultimately diagnosed as meningothelial meningioma.

**Case description:**

A 50-year-old woman presented with a 2-year history of painless, progressive visual impairment and a perceived altitudinal visual field defect in the left eye, with MRI revealing a retrobulbar T2-weighted hyperintense lesion of unclear nature in the setting of a recent diagnosis of breast carcinoma. Neuro-ophthalmic evaluation showed superonasal parapapillary retinoschisis, inferotemporal ganglion cell loss on OCT, and absolute superior scotoma on static perimetry, with preserved central visual acuity. To exclude metastatic disease and confirm the suspected diagnosis, an optic nerve sheath biopsy was performed, revealing WHO grade I meningothelial meningioma. Postoperatively, the visual field defect initially appeared enlarged. At the 1-week follow-up, there was reduced visual acuity and OCT findings consistent with a microvascular ischemic event, whereas the visual field defect returned to its preoperative pattern.

**Conclusion:**

This case highlights the diagnostic challenges of atypical retrobulbar optic nerve sheath lesions in the context of concurrent malignancy and underscores the feasibility and value of tissue diagnosis in selected patients to guide management.

## Introduction

The optic nerve sheath meningioma (ONSM) is an uncommon, benign neoplasm arising from the meningothelial (arachnoid cap) cells surrounding the optic nerve; it accounts for approximately 1–2% of all meningiomas and approximately 2% of orbital tumors, with a pronounced predilection for middle-aged women (1, 2). Typically, patients present with a slowly progressive, painless visual loss, frequently accompanied by optic disk changes (swelling or later atrophy), optic atrophy, or optociliary shunt vessels, and sometimes proptosis or restricted eye movements (3–5). Neuroimaging, particularly magnetic resonance imaging (MRI), is the diagnostic cornerstone and has largely obviated the need for biopsy in classical cases (6). On MRI, typical ONSMs are isointense to gray matter on T1- and T2-weighted images and show vivid homogeneous enhancement on contrast-enhanced, fat-suppressed T1 sequences (6, 7). The tumor commonly manifests as a circumferential ‘tube’ enveloping the optic nerve, the so-called “en plaque” growth pattern (most frequent, ~60–65%), but exophytic or fusiform patterns have also been described (4). As these lesions are normally found deeper in the eye socket, cysts are occasionally found between the optic bulb and the anterior margin of the meningioma (8, 9). On axial post-contrast images, the classical “tram-track” sign, that is, contrast enhancement of the sheath around a non-enhancing optic nerve, is typical; on coronal images, a “doughnut” or “target” configuration may be seen (4, 7). Given this well-characterized radiologic profile, small perineural or perioptic cysts, inflammatory sheath thickening, optic nerve glioma, lymphoma, or metastatic disease are usually considered in the differential diagnosis, especially when calcification is absent or when the pattern deviates from the classic (3, 4, 6). Our case deviates from the typical presentation in several respects, such as the highly localized lesion immediately posterior to the globe and adjacent to the optic nerve, as well as the absence of radiological evidence of tubular expansion along the intraorbital optic nerve sheath. Given that classical MRI features are often relied upon to establish the diagnosis without histology, such an atypical presentation may pose a diagnostic challenge, especially when metastasis due to concurrent malignancies cannot be ruled out.

### Case description

A 50-year-old woman presented to our neuro-ophthalmology department with a history of painless, slowly progressive visual impairment in the left eye over approximately 2 years. She reported noticing a scotoma in the superior visual field of the left eye. However, no objective ophthalmologic records were available. No episodes of sudden vision loss were reported.

**Figure 1:**
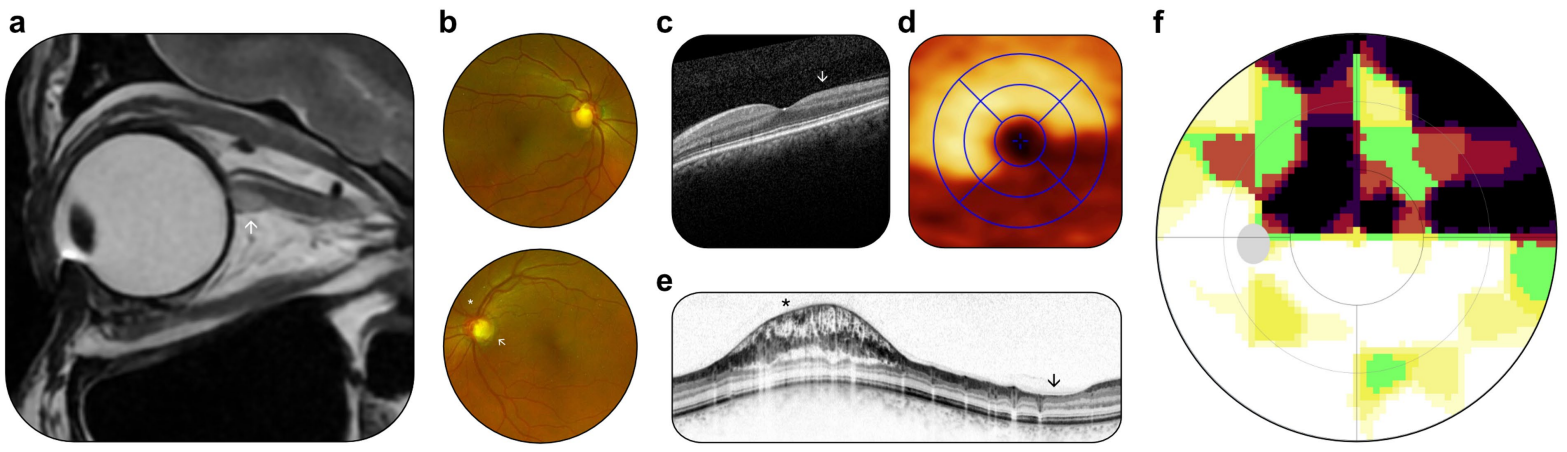
Preoperative outpatient examination. **(a)** Sagittal T2-weighted MRI scan, lesion (marked by white arrowhead) directly posterior to the optic bulb. **(b)** Fundus photography, visible optic disk rim notch inferotemporal of the left eye (marked by white arrowhead), and parapapillary retinoschisis (marked by black asterisk). **(c)** OCT scan, retinal cross-section, reduction in ganglion cell volume (marked by white arrowhead). **(d)** OCT scan, *en face* view, reduction in ganglion cell volume in inferotemporal sectors. **(e)** OCT scan, retinal nerve fiber layer, parapapillary retinoschisis (marked by black asterisk), and focal retinal nerve fiber atrophy (marked by black arrowhead). **(f)** Static perimetry, upper hemifield scotoma, corresponding to ganglion cell layer (GCL) thinning on OCT.

Five months before presentation, an external MRI, specifically a sagittal (non-fat-saturated), high-resolution T2-weighted sequence (2 mm slice thickness), revealed a well-defined lenticular, round-oval lesion in the proximal outer part of the perioptic nerve sheath in a caudomedial position, measuring 3 × 5 mm, without correlation in the identically recorded sagittal T1-weighted fat-saturated sequence after contrast administration. In the coronal, fat-saturated T2-weighted sequence (3 mm), a hyperintense correlate was detected on only a single slice. As this finding was unusual for an ONSM, being focal, eccentric, and lacking reliable contrast enhancement, it raised suspicion of a metastatic process as a differential diagnosis (Figure 1a). At that stage, no referral to our department had occurred.

One month before presentation, the patient had been diagnosed with breast carcinoma, for which local surgery was performed around the time of her initial outpatient visit to our department. The patient then presented to our clinic, primarily on her own initiative. At the time of presentation, systemic staging had not revealed any metastatic disease.

Slit-lamp biomicroscopy demonstrated superonasal retinoschisis and notching of the inferotemporal neuroretinal rim (Figure 1b). The fovea centralis and peripheral retina were unremarkable. Optical coherence tomography (OCT) showed preservation of the foveal shape with evidence of inferotemporal ganglion cell loss (Figures 1c,d) and focal retinoschisis with defects in the peripapillary retinal nerve fiber layer (Figure 1e). Haag-Streit static perimetry revealed an absolute scotoma in the superior visual field of the left eye (Figure 1f), corresponding to the observed ganglion cell loss. Best-corrected visual acuity was 20/20 Snellen.

Following an extended discussion on weighting operative risk to diagnostic reward in a multidisciplinary tumor board, further diagnostic clarification was ultimately pursued in our neuro-ophthalmology department, as the patient wished and at the request of the radiation oncology department.

In a follow-up in-house repeated MRI of the orbit (without sagittal T2-weighted imaging), the findings could only be reproduced from the preliminary images. T1 post-contrast fat-suppressed imaging, diffusion-weighted imaging, as well as axial and coronal imaging, were performed and reviewed; no images clearly depicting the lesion could be obtained. Importantly, no restricted diffusion hyperintensity in close proximity to the lesion was identified.

After shared and informed decision-making with the patient, we opted for surgical intervention to obtain a biopsy to confirm the suspected diagnosis of ONSMs and exclude other etiologies, such as metastatic disease from the recently diagnosed breast carcinoma. Surgery was performed 1 month after the initial presentation. We used a surgical approach analogous to optic nerve sheath fenestration, involving temporary detachment of the medial rectus tendon and maximal abduction of the globe (for further reference, see Lagrèze, 2009) (10).

**Figure 2:**
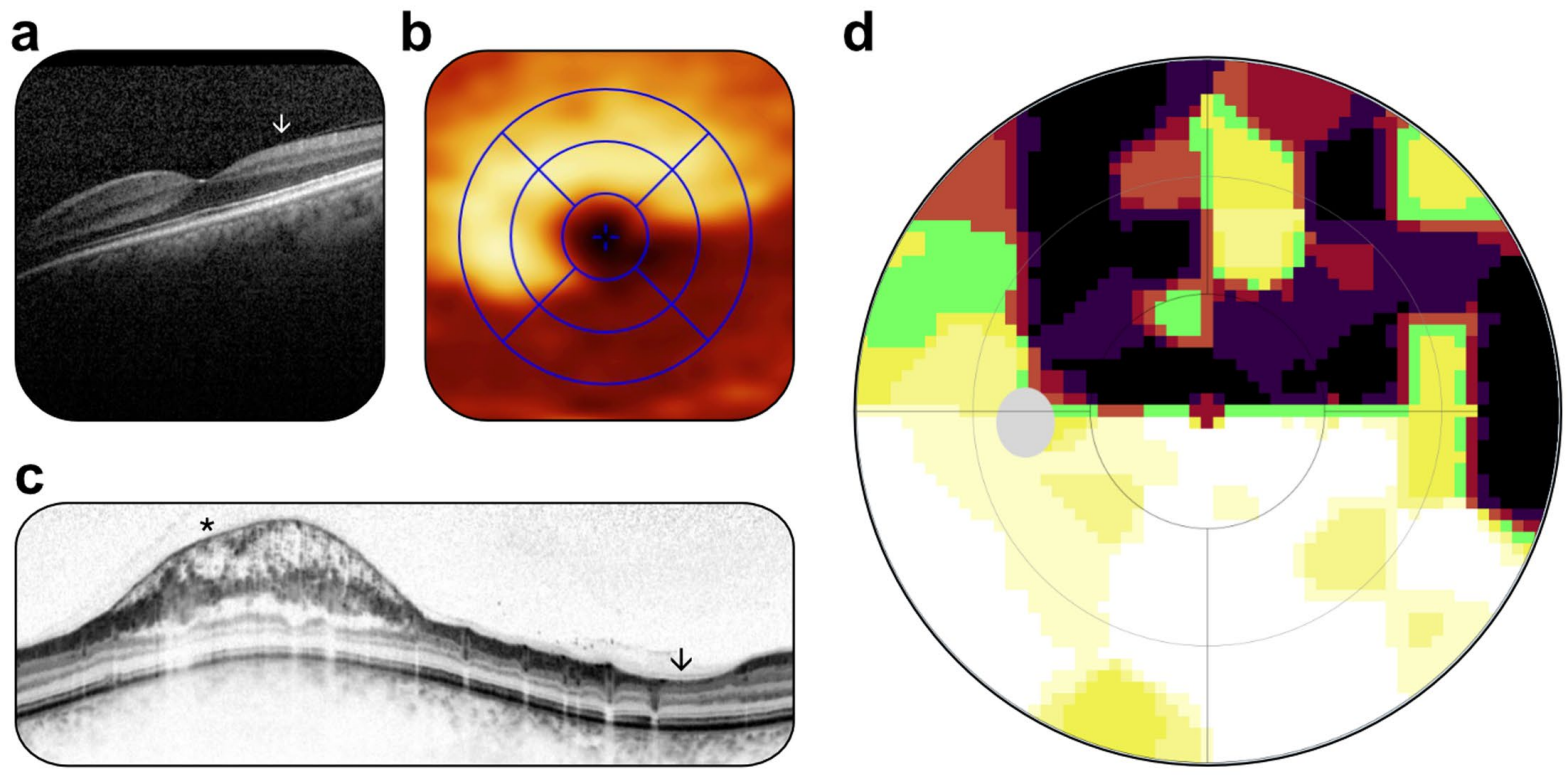
Postoperative inpatient examination. **(a)** OCT scan, retinal cross-section, reduction in ganglion cell volume (marked by white arrowhead), stable with preoperative status. **(b)** OCT scan, en face view, reduction in ganglion cell volume in inferotemporal sectors, stable compared with preoperative status. **(c)** OCT scan, retinal nerve fiber layer, parapapillary retinoschisis (marked by black asterisk), and focal retinal nerve fiber atrophy (marked by black arrowhead), stable compared with preoperative status. **(d)** Static perimetry, upper hemifield scotoma, slightly increased scotoma size, nasally accentuated, attributable to postoperative swelling.

The initial postoperative examination revealed no changes in the optic disk, fovea, and peripheral retina, corroborated by OCT. Visual acuity initially remained 20/20 Snellen. Minimal change in the upper hemifield scotoma on Haag-Streit static perimetry was attributed to postoperative edema (Figure 2).

**Figure 3:**
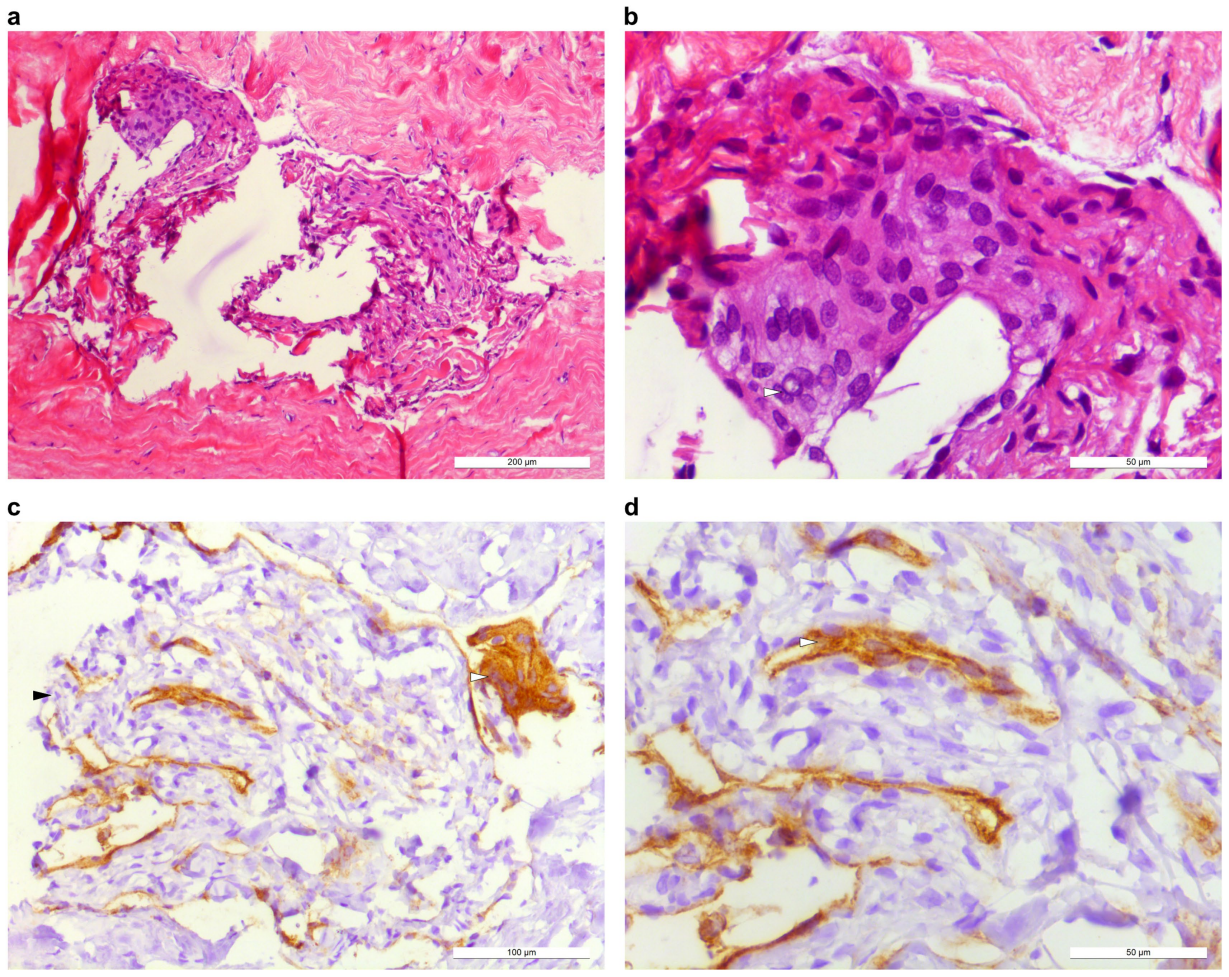
Histopathological work-up. **(a,b)** H&E staining, epithelioid cells with syncytia-like lobular growth pattern, eosinophilic cytoplasm, frequent nuclear clearing. **(c,d)** Positive EMA immunostaining in several tumor cells.

Surgically obtained material was subsequently histopathologically analyzed. Two small, gray tissue fragments with a firm, elastic consistency were subjected to formalin fixation and paraffin embedding. Conventional histological specimens were prepared for H&E, PAS, and EvG. Additionally, the material was treated with antibodies against PanCK, EMA, vimentin, synaptophysin, and Ki-67 (MIB-1). Within the fully examined biopsy, small fragments of a uniformly leptomeningeal differentiated tumor with dural invasion and typical immunoreactivity for vimentin and focal EMA expression were detected. Mitotic figures or increased proliferative activity (Ki-67) were not detected. Due to the immunonegativity of PanCk, carcinoma metastasis could be ruled out. Histopathological analysis, therefore, confirmed the diagnosis of meningioma, CNS WHO grade I (subtype: meningothelial meningioma) (Figure 3).

**Figure 4:**
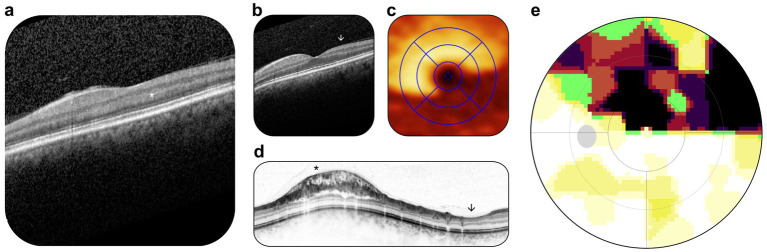
Postoperative outpatient examination. **(a)** OCT scan, hyperreflectivity of the outer plexiform and outer nuclear layers (marked by white asterisk). **(b)** OCT scan, retinal cross-section, reduction in ganglion cell volume (marked by the white arrowhead), stable compared with preoperative status. **(c)** OCT scan, en face view, reduction in ganglion cell volume in inferotemporal sectors, stable compared with preoperative status. **(d)** OCT scan, retinal nerve fiber layer, parapapillary retinoschisis (marked by black asterisk) and focal retinal nerve fiber atrophy (marked by black arrowhead), stable compared with preoperative status. **(e)** Static perimetry, upper hemifield scotoma, slightly increased scotoma size, stable compared with preoperative status.

At the 1-week postoperative follow-up, visual acuity had decreased to 20/32 Snellen. OCT demonstrated hyperreflectivity at the outer plexiform and outer nuclear layers, consistent with a microvascular ischemic event (Figure 4a). Parapapillary retinoschisis had regressed, while static perimetry confirmed that the upper hemifield scotoma had regressed to preoperative status (Figures 4b–e).

A subsequent multidisciplinary tumor board recommended adjuvant radiotherapy, which is currently being planned in cooperation with the Department of Radiation Oncology.

## Discussion

ONSMs classically present as circumferential, longitudinally extensive lesions that envelop the optic nerve along a considerable intraorbital course. Their characteristic imaging appearance, including tubular sheath thickening, homogeneous enhancement, and tram-track or doughnut configurations, typically enables confident diagnosis without histopathologic confirmation (4, 6). In contrast, the lesion in our patient exhibited a distinctly atypical morphology: a small, focal mass located immediately posterior to the globe at the optic nerve–globe junction, without evidence of the expected longitudinal sheath involvement or classic radiologic hallmarks. To our knowledge, such a highly localized, anteriorly situated ONSMs directly abutting the posterior pole of the globe has rarely, if ever, been described in the literature.

This atypical configuration introduced substantial diagnostic ambiguity, particularly in the context of recently diagnosed breast carcinoma. Orbital metastases may mimic benign lesions on MRI, and the radiographic differential includes cystic lesions, periorbital inflammation, and early metastatic disease. Given this diagnostic uncertainty, reliance on imaging alone would carry the risk of misclassification, potentially delaying appropriate therapy. Our case, therefore, underscores an important, albeit selective, role for surgical biopsy in the diagnostic workup of atypical optic nerve sheath lesions. While biopsy has largely fallen out of routine practice in ONSMs because of its classical radiologic signature and the risk of visual compromise, our experience demonstrates that, in carefully selected patients—especially those with concurrent malignancy, atypical imaging features, or lesions located anteriorly in the orbit—tissue sampling represents both a feasible and clinically decisive approach.

Importantly, the open incision biopsy approach in this case proved technically practical despite the lesion’s anterior location and proximity to the optic nerve and globe. In contrast, alternative biopsy techniques, including fine-needle aspiration biopsy and core needle biopsy (11, 12), were considered but ultimately deemed unsuitable due to the challenging anatomical location.

The postoperative course was initially stable, and although the patient later developed a central microvascular ischemic event, this complication did not alter the overall diagnostic or therapeutic trajectory. The histopathologic confirmation of a WHO grade I meningothelial meningioma immediately clarified management, allowing the multidisciplinary team to proceed confidently with adjuvant radiotherapy, an evidence-based treatment for ONSMs that aims to preserve remaining vision and prevent progression.

### Patient perspective

The patient demonstrated a positive affect throughout the course of care and expressed satisfaction with the treatment received. They actively engaged in discussions regarding the treatment plan, demonstrated understanding of the proposed interventions, and voiced agreement with their implementation, including advocating for a biopsy to obtain a definitive diagnosis. No concerns or objections were reported, and the patient consistently conveyed confidence in the clinical decisions made.

## Conclusion

This case demonstrates that ONSMs can present in atypical, highly localized forms that challenge standard imaging-based diagnosis, particularly in patients with concurrent systemic malignancy. In such situations, a carefully selected surgical biopsy can provide decisive diagnostic clarity and enable timely, vision-preserving management.

## Data Availability

The original contributions presented in the study are included in the article/supplementary material, further inquiries can be directed to the corresponding author.
